# 4-Benzyl-3-(2-fur­yl)-1*H*-1,2,4-triazole-5(4*H*)-thione hemihydrate

**DOI:** 10.1107/S1600536808012361

**Published:** 2008-05-03

**Authors:** Muhammad Zareef, Rashid Iqbal, Masood Parvez

**Affiliations:** a Department of Chemistry, Quaid-i-Azam University, Islamabad 45320, Pakistan; b Department of Chemistry, The University of Calgary, 2500 University Drive NW, Calgary, Alberta, Canada T2N 1N4

## Abstract

In the asymmetric unit of the title compound, C_13_H_11_N_3_OS·0.5H_2_O, there are two independent mol­ecules of 4-benzyl-3-(2-fur­yl)-1*H*-1,2,4-triazole-5(4*H*)-thione and a water mol­ecule of hydration. The conformation of the two organic  mol­ecules is slightly different, the dihedral angles formed by the furyl and triazole rings being 5.63 (15) and 17.66 (13)°. The water mol­ecule of hydration links three adjacent triazole mol­ecules to form a cluster *via* inter­molecular O—H⋯S, N—H⋯S and N—H⋯O hydrogen bonds, generating a 10-membered ring of graph set *R* ^3^_3_(10). The crystal structure is further stabilized by intra- and inter­molecular C—H⋯S, C—H⋯O and C—H⋯N hydrogen bonds and by π–π stacking inter­actions involving the furyl and triazole rings of centrosymmetrically related mol­ecules, with a centroid–centroid separation of 3.470 (2) Å.

## Related literature

For related literature, see: Ahmad *et al.* (2001 ▶); Altman & Solomost (1993 ▶); Chai *et al.* (2003 ▶); Dege *et al.* (2004 ▶); Hashimoto *et al.* (1990 ▶); Kanazawa *et al.* (1988 ▶); Öztürk *et al.* (2004 ▶); Yıldırım *et al.* (2004 ▶); Bernstein *et al.* (1995 ▶); Etter (1990 ▶).
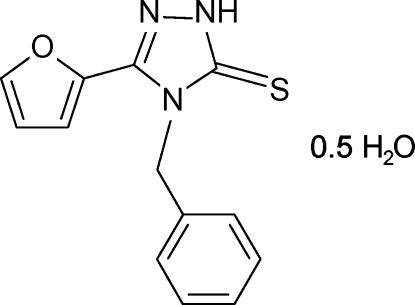


## Experimental

### 

#### Crystal data

C_13_H_11_N_3_OS·0.5H_2_O
*M* *_r_* = 266.33
Triclinic, 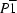

*a* = 6.082 (2) Å
*b* = 12.069 (4) Å
*c* = 17.818 (5) Å
α = 92.43 (2)°
β = 94.35 (2)°
γ = 103.83 (2)°
*V* = 1263.9 (7) Å^3^
*Z* = 4
Mo *K*α radiation
μ = 0.25 mm^−1^
*T* = 173 (2) K
0.18 × 0.16 × 0.04 mm

#### Data collection

Nonius KappaCCD diffractometer
Absorption correction: multi-scan (*SORTAV*; Blessing, 1997 ▶) *T*_min_ = 0.956, *T*_max_ = 0.990
10729 measured reflections
5735 independent reflections
4365 reflections with *I* > 2σ(*I*)
*R*_int_ = 0.031

#### Refinement

*R*[*F* ^2^ > 2σ(*F* ^2^)] = 0.039
*wR*(*F* ^2^) = 0.096
*S* = 1.03
5735 reflections
347 parameters
H atoms treated by a mixture of independent and constrained refinement
Δρ_max_ = 0.22 e Å^−3^
Δρ_min_ = −0.24 e Å^−3^

### 

Data collection: *COLLECT* (Hooft, 1998 ▶); cell refinement: *DENZO* (Otwinowski & Minor, 1997 ▶); data reduction: *SCALEPACK* (Otwinowski & Minor, 1997 ▶); program(s) used to solve structure: *SHELXS97* (Sheldrick, 2008 ▶); program(s) used to refine structure: *SHELXL97* (Sheldrick, 2008 ▶); molecular graphics: *ORTEP-3 for Windows* (Farrugia, 1997 ▶); software used to prepare material for publication: *SHELXL97*.

## Supplementary Material

Crystal structure: contains datablocks global, I. DOI: 10.1107/S1600536808012361/rz2207sup1.cif

Structure factors: contains datablocks I. DOI: 10.1107/S1600536808012361/rz2207Isup2.hkl

Additional supplementary materials:  crystallographic information; 3D view; checkCIF report

## Figures and Tables

**Table 1:** Hydrogen-bond geometry (Å, °)

*D*—H⋯*A*	*D*—H	H⋯*A*	*D*⋯*A*	*D*—H⋯*A*
O3—H3*A*⋯N5^i^	0.85 (2)	2.08 (2)	2.906 (2)	165 (2)
N1—H1*A*⋯S2^ii^	0.88 (2)	2.47 (2)	3.267 (2)	151 (2)
N4—H4*A*⋯O3^iii^	0.89 (2)	1.81 (2)	2.697 (2)	174 (2)
C7—H7*A*⋯N2^iv^	0.99	2.60	3.304 (3)	128
C7—H7*B*⋯O1^iv^	0.99	2.59	3.440 (2)	144
O3—H3*B*⋯S1	0.85 (2)	2.50 (2)	3.320 (2)	162 (2)
C7—H7*A*⋯S1	0.99	2.74	3.237 (2)	112
C9—H9⋯N3	0.95	2.56	2.893 (2)	101
C26—H26⋯N6	0.95	2.58	2.903 (3)	100
C20—H20*B*⋯S2	0.99	2.78	3.214 (2)	107

Symmetry codes: (i) 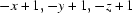; (ii) 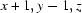; (iii) 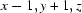; (iv) 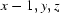.

**Fig. 1.**
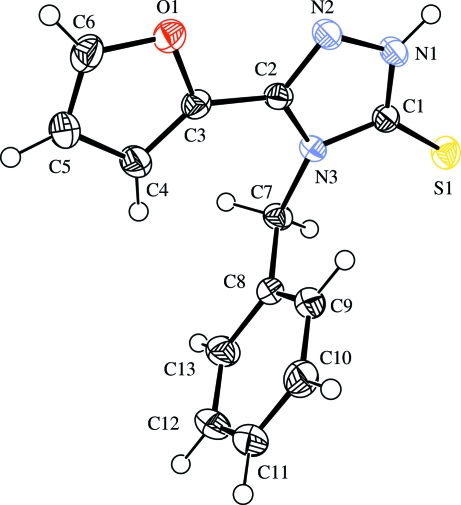
ORTEP-3 (Farrugia, 1997) drawing of molecule A with displacement ellipsoids plotted at the 50% probability level.

**Fig. 2.**
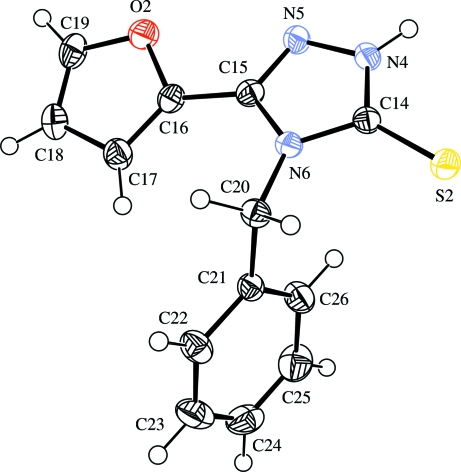
ORTEP-3 (Farrugia, 1997) drawing of molecule B with displacement ellipsoids plotted at the 50% probability level.

**Fig. 3.**
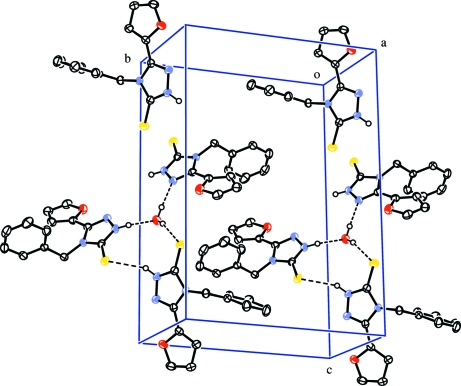
Packing diagram of the title compound showing intermolecular hydrogen bonds as dashed lines. H atoms not involved in H bonding are omitted for clarity.

## supplementary crystallographic information

### Comment

Recently, much attention has been focused on disubstituted 1,2,4-triazole derivatives for their broad-spectrum biological and pharmacological activities, such as fungicidal, herbicidal, anticonvulsant, antitumoral and inhibition of cholesterol (Chai *et al.*, 2003; Kanazawa *et al.*, 1988; Hashimoto *et al.*, 1990). In addition, they have many applications in agriculture domain (Altman & Solomost, 1993). In this paper, we report the synthesis and crystal structure of the title compound.

The asymmetric unit of the title compound is composed of two independent molecules (hereafter called A and B) depicted  in  Fig. 1 and 2, respectively, and a water molecule of hydration. The furyl and triazole rings in molecule A are substantially planar (maximum deviation 0.0586 (13) Å for atom N2), with the S1 atom 0.1980 (16) Å out of this plane; the mean-planes of the furyl and triazole form a dihedral angle of 5.63 (15)°. The corresponding furyl and triazole rings in molecule B are far from planar with atoms O2 and C17 deviating from the plane by 0.2571 (11) and -0.1633 (15) Å, respectively. The mean-planes of the five-membered rings in molecule B form an angle 17.66 (13)°. In both molecules, the benzyl rings are oriented at 80.72 (4) and 80.70 (4)°, from the planes formed by the ten atoms of the furyl and triazole rings in the molecules A and B, respectively. Bond distances and angles in the two molecules agree well with each other. Similar bond distances and bond angles have been reported in compounds closely related to the title compound, *e.g.*, 4-chlorophenyl analogue (Öztürk *et al.*, 2004), 4-methoxyphenyl analogue (Yıldırım *et al.*, 2004) and 4-*p*-tolyl (Dege *et al.*, 2004); in all these compounds, the mean-planes of the phenyl rings and those of the furyl and triazole rings lie close to right angles. The water molecule of hydration links three adjacent molecules of the title compound to form a cluster *via* intermolecular hydrogen bonds (Fig. 3, Table 1), forming a 10-membered ring of graph set R^3^_3_(10) (Etter, 1990; Bernstein *et al.*, 1995). Nonconventional intermolecular C—H···N and C—H···O H-bonds are also present in addition to intramolecular C—H···S and C—H···N hydrogen interactions (Table 1). The crystal structure is further stabilized by π-π stacking interactions involving centrosymmetrically related furyl and triazole rings at (x, y, z) and (1-x, -y, -z) with a centroid-centroid separation of 3.470 (2) Å.

### Experimental

The title compound was prepared from the corresponding thiosemicarbazide by following the reported procedure (Ahmad *et al.*, 2001). 4-Benzyl-1-(2-furoyl)thiosemicarbazide (10 mmol) was dissolved in an aqueous 4 N sodium hydroxide solution (50 ml). The solution was heated to reflux for 7 h, cooled and filtered. The filtrate was acidified to pH of 4–5, with 4 N hydrochloric acid. The solid crude product was filtered off, washed with water and recrystallized from aqueous ethanol (60%). Crystals of the title compound were grown by slow evaporation of an ethanol solution over 11 days at room temperature (yield 81%).

### Refinement

Though all the H atoms could be distinguished in the difference Fourier map the H-atoms bonded to C-atoms were included at geometrically idealized positions and refined in the riding-model approximation with the following constraints: benzyl/furyl and methylene C—H distances were set to 0.95 and 0.99 Å, respectively; in all these instances *U*_iso_(H) = 1.2 *U*_eq_(C). H-atoms bonded to N– and water of hydration were located from a difference Fourier map and were allowed to refine with *U*_iso_ = 1.2 times *U*_eq_ of the atoms to which they were bonded. The final difference map was free of any chemically significant feature.

### Crystal data

**Table:**

C_13_H_11_N_3_OS·0.5H_2_O	*Z* = 4
*M**_r_* = 266.33	*F*_000_ = 556
Triclinic, *P*1	*D*_x_ = 1.400 Mg m^−^^3^
Hall symbol: -P 1	Melting point = 458–459 K
*a* = 6.082 (2) Å	Mo *K*α radiation λ = 0.71073 Å
*b* = 12.069 (4) Å	Cell parameters from 10729 reflections
*c* = 17.818 (5) Å	θ = 3.0–27.5º
α = 92.43 (2)º	µ = 0.25 mm^−^^1^
β = 94.35 (2)º	*T* = 173 (2) K
γ = 103.83 (2)º	Plate, colourless
*V* = 1263.9 (7) Å^3^	0.18 × 0.16 × 0.04 mm

### Data collection

**Table:**

Nonius KappaCCD diffractometer	5735 independent reflections
Radiation source: fine-focus sealed tube	4365 reflections with *I* > 2σ(*I*)
Monochromator: graphite	*R*_int_ = 0.031
*T* = 173(2) K	θ_max_ = 27.5º
ω and φ scans	θ_min_ = 3.0º
Absorption correction: Multi-scan(SORTAV; Blessing, 1997)	*h* = −7→7
*T*_min_ = 0.956, *T*_max_ = 0.990	*k* = −15→15
10729 measured reflections	*l* = −23→23

### Refinement

**Table:**

Refinement on *F*^2^	Hydrogen site location: inferred from neighbouring sites
Least-squares matrix: full	H atoms treated by a mixture of independent and constrained refinement
*R*[*F*^2^ > 2σ(*F*^2^)] = 0.039	*w* = 1/[σ^2^(*F*_o_^2^) + (0.0384*P*)^2^ + 0.468*P*] where *P* = (*F*_o_^2^ + 2*F*_c_^2^)/3
*wR*(*F*^2^) = 0.096	(Δ/σ)_max_ = 0.001
*S* = 1.03	Δρ_max_ = 0.22 e Å^−^^3^
5735 reflections	Δρ_min_ = −0.24 e Å^−^^3^
347 parameters	Extinction correction: SHELXL97 (Sheldrick, 2008), Fc^*^=kFc[1+0.001xFc^2^λ^3^/sin(2θ)]^-1/4^
Primary atom site location: structure-invariant direct methods	Extinction coefficient: 0.012 (2)
Secondary atom site location: difference Fourier map	

### Special details

**Table:**

Geometry. All e.s.d.'s (except the e.s.d. in the dihedral angle between two l.s. planes) are estimated using the full covariance matrix. The cell e.s.d.'s are taken into account individually in the estimation of e.s.d.'s in distances, angles and torsion angles; correlations between e.s.d.'s in cell parameters are only used when they are defined by crystal symmetry. An approximate (isotropic) treatment of cell e.s.d.'s is used for estimating e.s.d.'s involving l.s. planes.
Refinement. Refinement of *F*^2^ against ALL reflections. The weighted *R*-factor *wR* and goodness of fit *S* are based on *F*^2^, conventional *R*-factors *R* are based on *F*, with *F* set to zero for negative *F*^2^. The threshold expression of *F*^2^ > σ(*F*^2^) is used only for calculating *R*-factors(gt) *etc*. and is not relevant to the choice of reflections for refinement. *R*-factors based on *F*^2^ are statistically about twice as large as those based on *F*, and *R*- factors based on ALL data will be even larger.

### Fractional atomic coordinates and isotropic or equivalent isotropic displacement parameters (Å^2^)

**Table:**

	*x*	*y*	*z*	*U*_iso_*/*U*_eq_	
S1	0.34044 (8)	0.05680 (4)	0.28003 (3)	0.03056 (13)	
S2	−0.20179 (8)	0.77953 (4)	0.25164 (2)	0.02763 (13)	
O1	0.8468 (2)	0.08820 (11)	−0.01137 (7)	0.0305 (3)	
O2	0.5602 (2)	0.82229 (11)	0.51122 (7)	0.0325 (3)	
O3	0.7588 (3)	0.03225 (11)	0.40424 (8)	0.0333 (3)	
H3A	0.752 (4)	0.0567 (19)	0.4491 (13)	0.040*	
H3B	0.633 (4)	0.0293 (19)	0.3796 (13)	0.040*	
N1	0.6822 (3)	0.01773 (13)	0.20087 (8)	0.0260 (3)	
H1A	0.725 (3)	−0.0312 (17)	0.2307 (11)	0.031*	
N2	0.7734 (3)	0.03375 (13)	0.13290 (8)	0.0268 (3)	
N3	0.4856 (2)	0.11614 (11)	0.14138 (8)	0.0213 (3)	
N4	0.0028 (3)	0.87457 (12)	0.38823 (8)	0.0247 (3)	
H4A	−0.085 (3)	0.9228 (17)	0.3954 (11)	0.030*	
N5	0.1725 (3)	0.86844 (12)	0.44183 (8)	0.0254 (3)	
N6	0.1475 (2)	0.74212 (11)	0.34577 (8)	0.0222 (3)	
C1	0.5050 (3)	0.06404 (14)	0.20819 (9)	0.0232 (4)	
C2	0.6504 (3)	0.09428 (14)	0.09778 (9)	0.0223 (4)	
C3	0.6860 (3)	0.13059 (14)	0.02235 (9)	0.0240 (4)	
C4	0.5984 (4)	0.19649 (17)	−0.02503 (10)	0.0371 (5)	
H4	0.4838	0.2354	−0.0155	0.044*	
C5	0.7116 (4)	0.19619 (17)	−0.09219 (11)	0.0393 (5)	
H5	0.6871	0.2351	−0.1361	0.047*	
C6	0.8591 (3)	0.13059 (16)	−0.08148 (10)	0.0331 (4)	
H6	0.9580	0.1156	−0.1173	0.040*	
C7	0.3061 (3)	0.17367 (14)	0.12078 (10)	0.0243 (4)	
H7A	0.1900	0.1564	0.1574	0.029*	
H7B	0.2319	0.1417	0.0705	0.029*	
C8	0.3862 (3)	0.30216 (14)	0.11841 (9)	0.0246 (4)	
C9	0.5982 (3)	0.36345 (15)	0.14907 (10)	0.0296 (4)	
H9	0.7004	0.3246	0.1729	0.035*	
C10	0.6630 (4)	0.48138 (16)	0.14531 (11)	0.0371 (5)	
H10	0.8090	0.5228	0.1665	0.045*	
C11	0.5153 (4)	0.53864 (17)	0.11072 (11)	0.0404 (5)	
H11	0.5599	0.6192	0.1079	0.048*	
C12	0.3028 (4)	0.47821 (17)	0.08038 (12)	0.0414 (5)	
H12	0.2006	0.5174	0.0570	0.050*	
C13	0.2381 (3)	0.36062 (16)	0.08393 (11)	0.0335 (4)	
H13	0.0918	0.3195	0.0627	0.040*	
C14	−0.0174 (3)	0.79991 (14)	0.32916 (9)	0.0223 (4)	
C15	0.2585 (3)	0.78665 (14)	0.41473 (9)	0.0233 (4)	
C16	0.4402 (3)	0.74833 (15)	0.45380 (9)	0.0242 (4)	
C17	0.5195 (3)	0.65286 (16)	0.44984 (10)	0.0303 (4)	
H17	0.4653	0.5884	0.4151	0.036*	
C18	0.7002 (3)	0.66805 (17)	0.50797 (10)	0.0321 (4)	
H18	0.7905	0.6157	0.5196	0.039*	
C19	0.7177 (3)	0.77051 (17)	0.54297 (11)	0.0332 (4)	
H19	0.8252	0.8028	0.5843	0.040*	
C20	0.2004 (3)	0.65628 (14)	0.29446 (9)	0.0239 (4)	
H20A	0.3674	0.6670	0.2974	0.029*	
H20B	0.1489	0.6693	0.2422	0.029*	
C21	0.0932 (3)	0.53434 (14)	0.31051 (9)	0.0226 (4)	
C22	0.1902 (3)	0.44799 (16)	0.28516 (10)	0.0307 (4)	
H22	0.3270	0.4668	0.2610	0.037*	
C23	0.0880 (4)	0.33463 (17)	0.29509 (11)	0.0389 (5)	
H23	0.1549	0.2760	0.2774	0.047*	
C24	−0.1094 (4)	0.30642 (17)	0.33038 (12)	0.0405 (5)	
H24	−0.1788	0.2285	0.3369	0.049*	
C25	−0.2063 (4)	0.39117 (18)	0.35627 (12)	0.0410 (5)	
H25	−0.3424	0.3719	0.3808	0.049*	
C26	−0.1044 (3)	0.50534 (16)	0.34637 (11)	0.0318 (4)	
H26	−0.1714	0.5637	0.3644	0.038*	

### Atomic displacement parameters (Å^2^)

**Table:**

	*U*^11^	*U*^22^	*U*^33^	*U*^12^	*U*^13^	*U*^23^
S1	0.0307 (3)	0.0369 (3)	0.0255 (2)	0.0094 (2)	0.00647 (18)	0.00482 (19)
S2	0.0285 (3)	0.0262 (2)	0.0270 (2)	0.00626 (19)	−0.00410 (18)	0.00176 (17)
O1	0.0316 (7)	0.0369 (7)	0.0265 (6)	0.0140 (6)	0.0061 (5)	0.0016 (5)
O2	0.0357 (8)	0.0306 (7)	0.0296 (7)	0.0091 (6)	−0.0090 (6)	−0.0012 (5)
O3	0.0415 (9)	0.0352 (7)	0.0271 (7)	0.0190 (7)	−0.0012 (6)	−0.0030 (6)
N1	0.0273 (8)	0.0293 (8)	0.0243 (7)	0.0114 (7)	0.0023 (6)	0.0072 (6)
N2	0.0280 (8)	0.0299 (8)	0.0256 (8)	0.0116 (7)	0.0040 (6)	0.0065 (6)
N3	0.0206 (8)	0.0218 (7)	0.0225 (7)	0.0071 (6)	0.0008 (6)	0.0029 (5)
N4	0.0268 (8)	0.0231 (7)	0.0253 (7)	0.0093 (6)	−0.0010 (6)	0.0007 (6)
N5	0.0266 (8)	0.0262 (7)	0.0237 (7)	0.0083 (6)	−0.0019 (6)	0.0009 (6)
N6	0.0233 (8)	0.0199 (7)	0.0232 (7)	0.0055 (6)	0.0011 (6)	0.0008 (6)
C1	0.0241 (9)	0.0213 (8)	0.0230 (8)	0.0044 (7)	−0.0010 (7)	0.0003 (7)
C2	0.0221 (9)	0.0212 (8)	0.0243 (8)	0.0072 (7)	0.0009 (7)	0.0005 (7)
C3	0.0236 (9)	0.0258 (9)	0.0235 (8)	0.0073 (7)	0.0040 (7)	−0.0004 (7)
C4	0.0512 (13)	0.0423 (11)	0.0282 (10)	0.0277 (10)	0.0126 (9)	0.0110 (8)
C5	0.0592 (14)	0.0384 (11)	0.0263 (10)	0.0194 (10)	0.0126 (9)	0.0103 (8)
C6	0.0382 (12)	0.0355 (10)	0.0256 (9)	0.0070 (9)	0.0097 (8)	0.0007 (8)
C7	0.0211 (9)	0.0257 (9)	0.0276 (9)	0.0093 (7)	0.0002 (7)	0.0019 (7)
C8	0.0304 (10)	0.0245 (9)	0.0210 (8)	0.0109 (8)	0.0022 (7)	0.0008 (7)
C9	0.0339 (11)	0.0281 (9)	0.0272 (9)	0.0108 (8)	−0.0036 (8)	0.0004 (7)
C10	0.0440 (12)	0.0290 (10)	0.0328 (10)	0.0016 (9)	−0.0062 (9)	−0.0021 (8)
C11	0.0609 (15)	0.0250 (10)	0.0350 (11)	0.0113 (10)	−0.0012 (10)	0.0037 (8)
C12	0.0549 (14)	0.0336 (11)	0.0405 (11)	0.0231 (10)	−0.0070 (10)	0.0053 (9)
C13	0.0327 (11)	0.0320 (10)	0.0371 (10)	0.0131 (9)	−0.0046 (8)	0.0007 (8)
C14	0.0213 (9)	0.0193 (8)	0.0262 (8)	0.0034 (7)	0.0038 (7)	0.0052 (7)
C15	0.0238 (9)	0.0227 (8)	0.0224 (8)	0.0037 (7)	0.0020 (7)	0.0022 (7)
C16	0.0238 (9)	0.0274 (9)	0.0207 (8)	0.0055 (7)	−0.0001 (7)	0.0013 (7)
C17	0.0305 (10)	0.0340 (10)	0.0280 (9)	0.0123 (8)	0.0009 (8)	−0.0012 (8)
C18	0.0279 (10)	0.0413 (11)	0.0308 (10)	0.0150 (9)	0.0016 (8)	0.0057 (8)
C19	0.0281 (10)	0.0427 (11)	0.0281 (9)	0.0090 (9)	−0.0058 (8)	0.0069 (8)
C20	0.0253 (9)	0.0244 (9)	0.0228 (8)	0.0068 (7)	0.0053 (7)	0.0005 (7)
C21	0.0246 (9)	0.0237 (8)	0.0202 (8)	0.0080 (7)	−0.0001 (7)	0.0011 (7)
C22	0.0390 (11)	0.0327 (10)	0.0248 (9)	0.0165 (9)	0.0043 (8)	0.0012 (7)
C23	0.0612 (15)	0.0280 (10)	0.0306 (10)	0.0201 (10)	−0.0055 (10)	−0.0015 (8)
C24	0.0527 (14)	0.0241 (9)	0.0384 (11)	0.0019 (9)	−0.0137 (10)	0.0062 (8)
C25	0.0339 (12)	0.0383 (11)	0.0485 (12)	0.0020 (9)	0.0050 (9)	0.0146 (10)
C26	0.0296 (11)	0.0277 (9)	0.0402 (11)	0.0091 (8)	0.0085 (8)	0.0049 (8)

### Geometric parameters (Å, °)

**Table:**

S1—C1	1.676 (2)	C8—C9	1.382 (3)
S2—C14	1.682 (2)	C8—C13	1.394 (2)
O1—C6	1.370 (2)	C9—C10	1.389 (3)
O1—C3	1.372 (2)	C9—H9	0.9500
O2—C19	1.364 (2)	C10—C11	1.383 (3)
O2—C16	1.369 (2)	C10—H10	0.9500
O3—H3A	0.85 (2)	C11—C12	1.380 (3)
O3—H3B	0.85 (2)	C11—H11	0.9500
N1—C1	1.339 (2)	C12—C13	1.385 (3)
N1—N2	1.372 (2)	C12—H12	0.9500
N1—H1A	0.88 (2)	C13—H13	0.9500
N2—C2	1.309 (2)	C15—C16	1.440 (2)
N3—C1	1.380 (2)	C16—C17	1.353 (2)
N3—C2	1.380 (2)	C17—C18	1.425 (3)
N3—C7	1.460 (2)	C17—H17	0.9500
N4—C14	1.335 (2)	C18—C19	1.338 (3)
N4—N5	1.370 (2)	C18—H18	0.9500
N4—H4A	0.89 (2)	C19—H19	0.9500
N5—C15	1.314 (2)	C20—C21	1.509 (2)
N6—C14	1.374 (2)	C20—H20A	0.9900
N6—C15	1.378 (2)	C20—H20B	0.9900
N6—C20	1.461 (2)	C21—C26	1.382 (3)
C2—C3	1.447 (2)	C21—C22	1.390 (2)
C3—C4	1.349 (3)	C22—C23	1.386 (3)
C4—C5	1.425 (3)	C22—H22	0.9500
C4—H4	0.9500	C23—C24	1.375 (3)
C5—C6	1.340 (3)	C23—H23	0.9500
C5—H5	0.9500	C24—C25	1.377 (3)
C6—H6	0.9500	C24—H24	0.9500
C7—C8	1.514 (2)	C25—C26	1.394 (3)
C7—H7A	0.9900	C25—H25	0.9500
C7—H7B	0.9900	C26—H26	0.9500
			
C6—O1—C3	106.50 (14)	C12—C11—H11	120.1
C19—O2—C16	106.33 (14)	C10—C11—H11	120.1
H3A—O3—H3B	108 (2)	C11—C12—C13	120.22 (18)
C1—N1—N2	114.05 (14)	C11—C12—H12	119.9
C1—N1—H1A	126.0 (13)	C13—C12—H12	119.9
N2—N1—H1A	118.8 (13)	C12—C13—C8	120.44 (19)
C2—N2—N1	103.59 (14)	C12—C13—H13	119.8
C1—N3—C2	107.51 (14)	C8—C13—H13	119.8
C1—N3—C7	124.11 (14)	N4—C14—N6	104.29 (14)
C2—N3—C7	128.16 (14)	N4—C14—S2	128.50 (13)
C14—N4—N5	113.20 (14)	N6—C14—S2	127.21 (13)
C14—N4—H4A	126.2 (13)	N5—C15—N6	110.73 (15)
N5—N4—H4A	120.5 (13)	N5—C15—C16	123.67 (15)
C15—N5—N4	104.25 (14)	N6—C15—C16	125.58 (15)
C14—N6—C15	107.53 (14)	C17—C16—O2	109.93 (15)
C14—N6—C20	124.07 (14)	C17—C16—C15	135.42 (16)
C15—N6—C20	128.21 (14)	O2—C16—C15	114.60 (14)
N1—C1—N3	103.46 (14)	C16—C17—C18	106.48 (17)
N1—C1—S1	128.34 (13)	C16—C17—H17	126.8
N3—C1—S1	128.17 (13)	C18—C17—H17	126.8
N2—C2—N3	111.36 (15)	C19—C18—C17	106.48 (16)
N2—C2—C3	122.82 (15)	C19—C18—H18	126.8
N3—C2—C3	125.81 (15)	C17—C18—H18	126.8
C4—C3—O1	109.95 (15)	C18—C19—O2	110.79 (16)
C4—C3—C2	135.93 (17)	C18—C19—H19	124.6
O1—C3—C2	114.11 (15)	O2—C19—H19	124.6
C3—C4—C5	106.47 (17)	N6—C20—C21	114.33 (14)
C3—C4—H4	126.8	N6—C20—H20A	108.7
C5—C4—H4	126.8	C21—C20—H20A	108.7
C6—C5—C4	106.89 (17)	N6—C20—H20B	108.7
C6—C5—H5	126.6	C21—C20—H20B	108.7
C4—C5—H5	126.6	H20A—C20—H20B	107.6
C5—C6—O1	110.20 (16)	C26—C21—C22	119.00 (17)
C5—C6—H6	124.9	C26—C21—C20	122.02 (15)
O1—C6—H6	124.9	C22—C21—C20	118.90 (16)
N3—C7—C8	114.60 (14)	C23—C22—C21	120.24 (19)
N3—C7—H7A	108.6	C23—C22—H22	119.9
C8—C7—H7A	108.6	C21—C22—H22	119.9
N3—C7—H7B	108.6	C24—C23—C22	120.41 (19)
C8—C7—H7B	108.6	C24—C23—H23	119.8
H7A—C7—H7B	107.6	C22—C23—H23	119.8
C9—C8—C13	118.95 (17)	C23—C24—C25	119.95 (19)
C9—C8—C7	122.93 (15)	C23—C24—H24	120.0
C13—C8—C7	118.12 (16)	C25—C24—H24	120.0
C8—C9—C10	120.53 (17)	C24—C25—C26	119.9 (2)
C8—C9—H9	119.7	C24—C25—H25	120.1
C10—C9—H9	119.7	C26—C25—H25	120.1
C11—C10—C9	120.12 (19)	C21—C26—C25	120.52 (18)
C11—C10—H10	119.9	C21—C26—H26	119.7
C9—C10—H10	119.9	C25—C26—H26	119.7
C12—C11—C10	119.73 (18)		
			
C1—N1—N2—C2	1.32 (19)	C7—C8—C13—C12	179.87 (18)
C14—N4—N5—C15	−0.33 (19)	N5—N4—C14—N6	0.32 (19)
N2—N1—C1—N3	−1.84 (19)	N5—N4—C14—S2	−179.83 (13)
N2—N1—C1—S1	176.30 (13)	C15—N6—C14—N4	−0.18 (18)
C2—N3—C1—N1	1.59 (18)	C20—N6—C14—N4	−175.62 (14)
C7—N3—C1—N1	176.47 (14)	C15—N6—C14—S2	179.97 (13)
C2—N3—C1—S1	−176.56 (13)	C20—N6—C14—S2	4.5 (2)
C7—N3—C1—S1	−1.7 (2)	N4—N5—C15—N6	0.20 (19)
N1—N2—C2—N3	−0.20 (19)	N4—N5—C15—C16	−178.34 (16)
N1—N2—C2—C3	−179.37 (16)	C14—N6—C15—N5	−0.02 (19)
C1—N3—C2—N2	−0.90 (19)	C20—N6—C15—N5	175.17 (15)
C7—N3—C2—N2	−175.50 (15)	C14—N6—C15—C16	178.48 (16)
C1—N3—C2—C3	178.23 (16)	C20—N6—C15—C16	−6.3 (3)
C7—N3—C2—C3	3.6 (3)	C19—O2—C16—C17	0.2 (2)
C6—O1—C3—C4	0.6 (2)	C19—O2—C16—C15	177.89 (15)
C6—O1—C3—C2	−179.92 (15)	N5—C15—C16—C17	159.8 (2)
N2—C2—C3—C4	−175.6 (2)	N6—C15—C16—C17	−18.5 (3)
N3—C2—C3—C4	5.4 (3)	N5—C15—C16—O2	−17.1 (2)
N2—C2—C3—O1	5.1 (2)	N6—C15—C16—O2	164.55 (16)
N3—C2—C3—O1	−173.93 (15)	O2—C16—C17—C18	−0.2 (2)
O1—C3—C4—C5	−0.4 (2)	C15—C16—C17—C18	−177.2 (2)
C2—C3—C4—C5	−179.8 (2)	C16—C17—C18—C19	0.1 (2)
C3—C4—C5—C6	0.1 (2)	C17—C18—C19—O2	0.0 (2)
C4—C5—C6—O1	0.3 (2)	C16—O2—C19—C18	−0.1 (2)
C3—O1—C6—C5	−0.5 (2)	C14—N6—C20—C21	−98.91 (19)
C1—N3—C7—C8	111.39 (18)	C15—N6—C20—C21	86.6 (2)
C2—N3—C7—C8	−74.8 (2)	N6—C20—C21—C26	25.8 (2)
N3—C7—C8—C9	−13.9 (2)	N6—C20—C21—C22	−157.33 (16)
N3—C7—C8—C13	166.36 (16)	C26—C21—C22—C23	0.8 (3)
C13—C8—C9—C10	−0.3 (3)	C20—C21—C22—C23	−176.17 (16)
C7—C8—C9—C10	180.00 (17)	C21—C22—C23—C24	−0.3 (3)
C8—C9—C10—C11	0.0 (3)	C22—C23—C24—C25	−0.2 (3)
C9—C10—C11—C12	0.4 (3)	C23—C24—C25—C26	0.2 (3)
C10—C11—C12—C13	−0.5 (3)	C22—C21—C26—C25	−0.7 (3)
C11—C12—C13—C8	0.3 (3)	C20—C21—C26—C25	176.14 (17)
C9—C8—C13—C12	0.1 (3)	C24—C25—C26—C21	0.2 (3)

### Hydrogen-bond geometry (Å, °)

**Table:**

*D*—H···*A*	*D*—H	H···*A*	*D*···*A*	*D*—H···*A*
O3—H3A···N5^i^	0.85 (2)	2.08 (2)	2.906 (2)	165 (2)
N1—H1A···S2^ii^	0.88 (2)	2.47 (2)	3.267 (2)	151 (2)
N4—H4A···O3^iii^	0.89 (2)	1.81 (2)	2.697 (2)	174 (2)
C7—H7A···N2^iv^	0.99	2.60	3.304 (3)	128
C7—H7B···O1^iv^	0.99	2.59	3.440 (2)	144
O3—H3B···S1	0.85 (2)	2.50 (2)	3.320 (2)	162 (2)
C7—H7A···S1	0.99	2.74	3.237 (2)	112
C9—H9···N3	0.95	2.56	2.893 (2)	101
C26—H26···N6	0.95	2.58	2.903 (3)	100
C20—H20B···S2	0.99	2.78	3.214 (2)	107

Symmetry codes: (i) −*x*+1, −*y*+1, −*z*+1; (ii) *x*+1, *y*−1, *z*; (iii) *x*−1, *y*+1, *z*; (iv) *x*−1, *y*, *z*.
